# Bone Development and Disease in Infants

**DOI:** 10.3390/children9040519

**Published:** 2022-04-06

**Authors:** Vito Pavone

**Affiliations:** Department of General Surgery and Medical Surgical Specialties, Section of Orthopaedics and Traumatology, A.O.U. Policlinico Rodolico—San Marco, University of Catania, 95123 Catania, Italy; vitopavone@hotmail.com

The aim of this Editorial is to introduce the content of the present Special Issue, entitled “Bone Development and Disease in Infants”. Over the years, the orthopedic management of children affected by bone diseases has seen numerous changes, thanks to the continuous scientific confrontation and advances. It is therefore fundamental to keep on researching and exchanging ideas and results, aiming to give the best chances possible to our little patients. For this reason, this Special Issue represents a wonderful occasion since it provides a picture of today’s knowledge and creates a collective discussion on the hot topics of pediatric orthopedics. From trauma to congenital and developmental disease, to the analysis of normal bone development, this Special Issue presents a selection of eleven outstanding articles, chosen carefully for our readers from numerous valid manuscripts. These articles are very interesting and constitute valid ground for everyone in the field.

The collection starts with the latest research works on a timeless topic: clubfoot. In the first communication, the analysis of the reliability of Pirani and Dimeglio scores in different medical figures (from residents to orthopedic surgeons) provides a picture of two solid evaluation methods that can be used widely by the medical community, especially for congenital talipes equinovarus [1]. Then, another selected manuscript regarding clubfoot analyzes sport ability during walking age following the Ponseti method: once again, it proves to be a valid therapeutic tool for these little patients [2].

Moving to another congenital orthopedic disease, developmental dysplasia of the hip, a group of colleagues underline the ineffectiveness of double diapering in its treatment [3]. In addition, it was decided to include a systematic review on this topic, in order to clarify DDH treatment options. This review analyzes dynamic and static splint differences, explaining the correct indication for the use of one or the other [4]. These first four articles question old standardized methods of diagnosis and treatment, confirming that the most used methods still represent the first choice.

In terms of more general topics, Moca et al. conducted an interesting retrospective comparative study on chronological age in different bone development stages by using lateral cephalometric radiographs and the cervical vertebral maturation method, adding useful information about bone growth and its stages [5]. Another classification was questioned in the article by Oh et al., where the duration of the Waldenström stage was used to evaluate early-onset Legg–Calvè–Perthes disease and its correlation with conservative treatment outcomes [6].

Moreover, two other reviews are included in this Special Issue. One is an analysis of sports and children with hemophilia that exhaustively assesses the actual trends in these patients’ care and everyday life [7], and the other review investigates the literature concerning the treatment of complex regional pain syndrome in children and adolescents, offering tools on how to handle these cases [8].

Between the different articles, a case report was selected for this collection, which is always useful to readers who can find ideas and stimuli for their own difficult cases. Zoccali et al. display a case of a brilliant proximal femur reconstruction after bone tumor resection in an infant patient [9].

It was only right to present a piece on pediatric trauma: one article studied the correlation between the dominant hand and supracondylar humerus fracture, suggesting that children probably tend to fall on their non-dominant hand to protect the dominant one [10].

The current pandemic could not go without discussion in this Special Issue, since our field has also been widely shaken by the ongoing SARS-CoV-2 pandemic that continues to affect our lives and those of our patients. Dibello et al. report differences in trauma in children during this unprecedented time, pointing out very interesting findings [11].

To conclude, I would like to thank the prestigious colleagues that helped me develop this Special Issue, analyzing a plethora of valid articles, and offering their best opinions. I hope this is going to be a pleasant read that can be helpful by adding useful information, widening readers’ knowledge, and fueling the collective discussion on these selected topics.

## References

[1] Pavone V., Vescio A., Culmone A., Caldaci A., Rosa P.L., Costarella L., Testa G. (2021). Interobserver Reliability of Pirani and Dimeglio Scores in the Clinical Evaluation of Idiopathic Congenital Clubfoot. Children.

[2] Pavone V., Vescio A., Caldaci A., Culmone A., Sapienza M., Rabito M., Canavese F., Testa G. (2021). Sport Ability during Walking Age in Clubfoot-Affected Children after Ponseti Method: A Case-Series Study. Children.

[3] De Pellegrin M., Damia C.M., Marcucci L., Moharamzadeh D. (2021). Double Diapering Ineffectiveness in Avoiding Adduction and Extension in Newborns Hips. Children.

[4] Pavone V., de Cristo C., Vescio A., Lucenti L., Sapienza M., Sessa G., Pavone P., Testa G. (2021). Dynamic and Static Splinting for Treatment of Developmental Dysplasia of the Hip: A Systematic Review. Children.

[5] Moca A.E., Vaida L.L., Moca R.T., Țuțuianu A.V., Bochiș C.F., Bochiș S.A., Iovanovici D.C., Negruțiu B.M. (2021). Chronological Age in Different Bone Development Stages: A Retrospective Comparative Study. Children.

[6] Oh H.-S., Sung M.-J., Lee Y.-M., Kim S., Jung S.-T. (2021). Does the Duration of Each Waldenström Stage Affect the Final Outcome of Legg–Calvé–Perthes Disease Onset before 6 Years of Age?. Children.

[7] Moretti L., Bizzoca D., Buono C., Ladogana T., Albano F., Moretti B. (2021). Sports and Children with Hemophilia: Current Trends. Children.

[8] Vescio A., Testa G., Culmone A., Sapienza M., Valenti F., Di Maria F., Pavone V. (2020). Treatment of Complex Regional Pain Syndrome in Children and Adolescents: A Structured Literature Scoping Review. Children.

[9] Zoccali C., Careri S., Attala D., Florio M., Milano G.M., Giordano M. (2021). A New Proximal Femur Reconstruction Technique after Bone Tumor Resection in a Very Small Patient: An Exemplificative Case. Children.

[10] Herdea A., Ulici A., Toma A., Voicu B., Charkaoui A. (2021). The Relationship between the Dominant Hand and the Occurrence of the Supracondylar Humerus Fracture in Pediatric Orthopedics. Children.

[11] Dibello D., Salvemini M., Amati C., Colella A., Graziano G., Vicenti G., Moretti B., Pederiva F. (2021). Trauma in Children during Lockdown for SARS-CoV-2 Pandemic. A Brief Report. Children.

